# miR-539-5p targets BMP2 to regulate Treg activation in B-cell acute lymphoblastic leukemia through TGF-β/Smads/MAPK

**DOI:** 10.3389/ebm.2024.10111

**Published:** 2024-02-13

**Authors:** Qingkai Dai, Rui Shi, Ge Zhang, Yuefang Wang, Lei Ye, Luyun Peng, Siqi Guo, Jiajing He, Hao Yang, Yongmei Jiang

**Affiliations:** ^1^ Department of Laboratory Medicine, West China Second University Hospital, Sichuan University, Chengdu, Sichuan, China; ^2^ Key Laboratory of Obstetric and Gynecological and Pediatric Diseases and Birth Defects of Ministry of Education, Chengdu, Sichuan, China

**Keywords:** miR-539-5p, BMP2, TGF-β, Treg, B-cell acute lymphoblastic leukemia

## Abstract

MicroRNAs (mRNAs) were believed to play an important role in cancers, and this study aimed to explore the mechanism of miRNA regulating Treg in B-cell acute lymphoblastic leukemia (B-ALL). Firstly, the differentially expressed miRNAs and target genes significantly associated with Tregs were screened out by high-throughput sequencing, and their enrichment pathways were analyzed. The binding relationship between miRNA and target genes was further verified, and the effects of miRNA on the proliferation and apoptosis of B-ALL Nalm-6 cells and Treg activation were analyzed. Results showed that differentially expressed miR-539-5p was significantly under-expressed, and its target gene BMP2 was significantly over-expressed in B-ALL, and significantly enriched in the TGF-β1 pathway. In addition, both miR-539-5p and BMP2 were significantly correlated with Treg activity in B-ALL. *In vitro* experiments further confirmed that miR-539-5p could directly target BMP2. The low expression of miR-539-5p in B-ALL significantly promoted BMP2 expression to promote the proliferation and inhibit apoptosis of Nalm-6 cells. Furthermore, the high expression of BMP2 in B-ALL could cooperate with TGF-β1 to promote the activation of human CD4^+^CD25^-^T cells to Treg, and significantly activate the TGF-β/Smads/MAPK pathway. *In vivo* experiments also confirmed that overexpression of miR-539-5p significantly inhibited BMP2 to suppress Treg activation and Smad1 and Smad2 phosphorylation, and finally inhibit the B-ALL process. In conclusion, miR-539-5p was significantly under-expressed in B-ALL and could target BMP2 to promote its expression, and the overexpressed BMP2 further promoted Treg activation in B-ALL by regulating TGF-β/Smads/MAPK pathway.

## Impact statement

Acute lymphoblastic leukemia (ALL) is a hematological malignancy characterized by abnormal proliferation of primary and juvenile lymphocytes in the bone marrow and peripheral blood. Exploring the pathogenesis of ALL is of great clinical significance for further improving the long-term survival rate and reducing the recurrence of ALL. Studies have shown that miRNA can be used for early clinical diagnosis and prognosis of tumors, and play an important role in ALL. In this study, high-throughput sequencing technology was used to screen the differentially expressed miRNAs of ALL, and the mechanism was explored based on the regulatory Treg. It was found that miR-539-5p was significantly under-expressed in ALL, and could target BMP2 to promote its expression. Overexpression of BMP2 further promoted the activation of Treg in ALL by regulating the TGF-β/Smads/MAPK pathway. The study can provide new ideas and methods for understanding the occurrence and development of ALL.

## Introduction

Acute lymphoblastic leukemia (ALL) is one of the most common hematological malignancies in children, accounting for about 20% of pediatric cancers [1]. It is mainly divided into B-cell ALL (B-ALL) and T-cell ALL (T-ALL), among which B-ALL accounts for 85% and T-ALL accounts for 15% [2]. In recent years, the 5-year survival rate of ALL is relatively high, which can reach 90% [3]. However, 20% of ALL are still at risk for relapse, and treatment outcomes after relapse are unsatisfactory and are the leading cause of death in ALL [4]. With the rise of immunotherapy, immunotherapies targeting ALL antigen-associated monoclonal antibodies and chimeric antigen receptor T cells have been shown to be effective against ALL and can significantly improve the prognosis of patients with relapsed/refractory ALL [5]. In addition, miRNAs have been described as modulators of a large number of different immune processes and have the potential to be key to the future development of immunotherapy [6]. Similarly, it is currently believed that the most important factors affecting the recurrence of ALL are the genetic and molecular biological abnormalities in the course of the disease [7]. Some studies have helped to demonstrate that specific miRNA gene expression signatures are associated with specific B- or T-ALL subpopulations. Further exploring the involvement of miRNA genes in the disease process of B-ALL alone or in the network, and clarifying the relationship between specific miRNA and the pathogenesis of B-ALL, will have important clinical significance for reducing the recurrence of B-ALL.

ALL, as cancer affecting lymphoid blood cells, originates primarily from B or T lymphoid progenitor cells [8]. T lymphocytes are an important member of cellular immunity in the body, which can be further divided into CD4^+^ T and CD8^+^ T cells [9]. Abnormal expression of CD4^+^ and CD8^+^ T cells has been confirmed in previous studies of patients with solid tumors [10, 11]. Regulatory T cells (Treg) are the immunosuppressive T cell subsets of CD4^+^ T cells, which can inhibit the activation and proliferation of effector T cells and the functions of primary and memory T lymphocytes, and participate in the maintenance of autoimmune homeostasis [12, 13]. Studies have found that Treg can suppress the anti-cancer immune response through a variety of immune regulatory pathways, leading to cancer evasion from immune surveillance [14]. The abnormal number and function of Treg are involved in a variety of solid cancers and hematological malignancies, and are correlated with the prognosis of cancers, which has important clinical value for the evaluation and prognosis of patients [15]. Moreover, studies have shown that Treg cells in B-ALL patients have an important relationship with immune deficiency [16], and the differential expression levels and regulation of Treg may affect the diagnosis and treatment of B-ALL. However, the specific mechanism of regulating Treg in B-ALL remains unclear and needs further study.

Therefore, in this study, high-throughput sequencing was used to analyze the whole transcriptome in peripheral blood mononuclear cells (PBMCs) of B-ALL, and the differentially expressed miR-539-5p and its target gene bone morphogenetic protein 2 (BMP2) were screened to be involved in Treg regulation in B-ALL. *In vitro* and *in vivo* experiments further confirmed the regulatory effects and the possible mechanism of the miR-539-5p/BMP2 regulatory network on Treg in B-ALL.

## Materials and methods

### Clinical sample collection

The high-throughput sequencing samples were collected from five newly diagnosed B-ALL children at West China Second University Hospital of Sichuan University, and five healthy children who underwent physical examination in the outpatient department of our hospital were selected as the control. The blood routine and various indexes of children in control group showed normal, with no previous history of hematological malignancy or family history. And there was no statistical difference in age or gender between the two groups. In addition, 46 B-ALL children and 42 healthy children matched by age and sex were included for follow-up study. The diagnostic criteria for B-ALL refer to Chinese Children Cancer Group (CCCG) ALL 2015 protocol (CCCG-ALL-2015). This study was approved by the Ethics Committee of West China Second University Hospital of Sichuan University, and all patients and their families agreed and signed informed consent.

### Isolation of PBMCs

Freshly 2 mL EDTA anticoagulated whole blood from B-ALL patients and healthy children were mixed with 2 mL PBS, and then slowly added to 3 mL of Ficoll-Paque density gradient media (cat. no. 17-5442-02; GE, United States). Centrifugation at room temperature at 400 g for 30 min, the top layer of plasma was removed, and the middle layer was washed with 3-fold volume of PBS. After centrifugation at 400 *g* for 10 min, the supernatant was discarded and the precipitation was PBMCs for subsequent study.

### Transcriptome sequencing analysis

Total RNA was extracted from PBMCs [17–19] by Trizol (cat. no. 15596026; Invitrogen, United States), and the integrity and concentration of RNA were examined using Agilent 2100 Bioanalyzer (Agilent, United States). The TruSeg Small RNA Sample Prep kit (cat. no. RS-200-0012; Illumina, United States) was used to obtain cDNA. The cDNA library was further constructed and enriched, and sequencing was performed on the Illumina platform. The original sequencing data were de-spliced and the quality-filtered sequences were de-processed. According to the human genome annotation, the abundance of the de-duplicated sequences was annotated. The characteristics and expression levels of miRNAs in PBMCs of B-ALL and controls were statistically analyzed. And DESeq software of R language (version 4.3.0) was used to analyze the differentially expressed miRNA and mRNA, while *p* < 0.05 and |log2FoldChange| >1.0 were defined as significantly differentially expressed mRNA or miRNA. Further, R language was used to cluster the differentially expressed miRNAs, predict the target genes, and perform functional enrichment analysis of the predicted target genes, including gene ontology (GO) and Kyoto Encyclopedia of Genes and Genomes (KEGG) analysis.

### Cell culture and transfection

Human B lymphoid leukemia cells Nalm-6 and human embryonic kidney cells H293-T were both purchased from Procell (Wuhan, China). H293-T cells were cultured in DMEM (Hyclone, United States) containing 10% FBS (CLARK BIOSCIENCE, United States) and 1% P/S (Hyclone, United States) and were used for double luciferase reporter assay. Nalm-6 cells were cultured in RPMI-1640 medium (Hyclone, United States) to analyze the role of miR-539-5p/BMP2 in the induction of Treg by Nalm-6 cells. Negative control-siRNA (NC-siRNA), siRNA targeting BMP2 (BMP2-siRNA), NC of pcDNA plasmid (pcDNA-NC), pc-DNA plasmid containing BMP2 sequence (pcDNA-BMP2), NC-mimic and miR-539-5p-mimic (all synthesized or purchased from Guangzhou Ribobio, China) were transfected into Nalm-6 cell by Lipofectamine 2000 (Beyotime, China).

### Double luciferase reporter assay

The binding sites of miR-539-5p and BMP2 were predicted by online software Targetscan^1^, and the BMP2 wild-type (BMP2-WT) and mutant sequence (BMP2-Mut) were constructed and cloned into the dual luciferase reporter pmirGLO-UTR vector. H293-T cells were co-transfected with the reporter plasmid and NC-mimic or miR-539-5p-mimic for 48 h by Lipofectamine 2000. Firefly luciferase and renilla luciferase values were determined and luciferase activity was calculated using the dual luciferase reporter kit (Promega Corporation, United States).

### Cell activity analysis

The proliferation activity of Nalm-6 cells was analyzed by Cell Counting Kit-8 (CCK8). After different treatments, Nalm-6 cells were incubated with 10 μL CCK8 detection reagent (cat. no. C0037; Beyotime, China) for 1 h. The absorbance was measured at 450 nm and the cell viability was calculated.

### Apoptosis detection

Apoptosis was detected by Annexin V-FITC/PI double staining. Nalm-6 cells with different transfection were collected, resuspended with 500 μL Binding Buffer, stained with 5 μL Annexin V-FITC, and 5 μL PI (cat. no. C1062S; Beyotime, China) for 15 min at room temperature under darkness, and then analyzed by flow cytometer (BD FACSCanto, United States).

### Sorting of CD4^+^CD25^-^T cells in PBMCs

The human CD4 T lymphocyte enrichment kit (cat. no. 557939; BD, United States) was used to enrich CD4^+^T cells in PBMCs of normal healthy subjects according to the manufacture instructions. Then, the number of enriched CD4^+^T lymphocytes was counted and washed by 1×BD IMag buffer (cat. no. 552362; BD, United States), and the BD IMag anti-human CD25 magnetic particle-DM (cat. no. 558005; BD, United States) was added to incubate for 30 min at room temperature. After bringing the BD IMag-particle labeling volumes up to 1–8 × 10^7^ cells/mL with 1 × BD IMag buffer, the tubes were immediately placed on a cell separation magnet (cat. no. 552311; BD, United States) to incubate at room temperature for 9 min. Finally, carefully aspirate the supernatant, which contains the negative fraction (CD4^+^CD25^-^T cells).

### Treg induction *in vitro*

Primary peripheral blood CD4^+^CD25^-^T cells were cultured in the RPMI-1640 medium containing anti-CD3 (5 μg/mL), anti-CD28 (5 μg/mL), IL-2 (100 U/mL) and retinoic acid (10 nM) at 37°C and 5% CO_2_ for 5 days for routine Treg induction. To analyze the role of miR-539-5p in Nalm-6 cell-induced Treg activation, subsequent experiments were divided into eight groups: control group, Nalm-6 cell supernatant group (NC), miR-539-5p-mimic transfected cell supernatant group (miR-539-5p-mimic), BMP2-siRNA transfected cell supernatant group (BMP2-siRNA), TGF-β1 positive control group (TGF-β1), TGF-β1+NC, TGF-β1+miR-539-5p-mimic, TGF-β1+BMP2-siRNA. The control group was treated by the routine induction method. Groups containing TGF-β1 were supplemented with 10 ng/mL TGF-β1. The NC, miR-539-5p-mimic and BMP2-siRNA groups were added with the culture supernatant of Nalm-6 cells transfected with NC, miR-539-5p-mimic and BMP2-siRNA at 1:1, respectively.

### Treg cell ratio analysis

The proportion of Treg in peripheral blood of B-ALL patients was characterized by CD3^+^CD4^+^CD25^+^CD127^-^ in this study. Briefly, 5 μL fluorescein-labeled monoclonal antibodies FITC-CD3 (cat. no. 11-0037-42; Invitrogen, United States), PerCP-Cy5.5-CD4 (cat. no. 45-0049-42; Invitrogen), PE-CD25 (cat. no. 12-0259-80; Invitrogen) and APC-CD127 (cat. no. 17-1278-42; Invitrogen) were added to 100 μL anticoagulant blood samples, and incubated at room temperature for 30 min away from light. Then, 2,000 μL of FACS Lysing Solution (BD, United States) was added and incubated at room temperature and away from light for 10 min. After washing and suspension with PBS, flow cytometer was used for detection. In addition, the proportion of Foxp3^+^ Treg in CD4^+^CD25^-^T cells after induction was also measured. The cells with different treatments were incubated with 5 μL FITC-labeled CD4 antibody (cat. no. 11-0041-82; Invitrogen) and APC-CD25 antibody (cat. no. MA5-16224; Invitrogen) at 4°C for 30 min. After washing with PBS, the membrane-breaking fixative was incubated at 4°C for 1 h. Then, 5 μL PE-Foxp3 antibody (cat. no. 12-4774-42; Invitrogen) was incubated at 4°C for 1 h. The proportion of Foxp3^+^Treg cells was detected by flow cytometer.

### Proliferation activity of CD4^+^CD25^-^T cells

The isolated CD4^+^CD25^-^T cells were induced at 37°C and 5% CO_2_ for 5 days according to different groups. After centrifugation, the cells were incubated at 37°C for 20 min with 2 μM carboxyfluorescein succinimidyl ester (CFSE) staining solution (cat. no. C0051; Beyotime, China), neutralized and washed twice with RPMI-1640 containing FBS. CFSE was detected by flow cytometry, and the proliferation rate was calculated.

### Animals experimental design

Eighteen NOD/SCID mice, weighing 18–20 g and aged 4–5 weeks, were purchased from Chengdu Dossy experimental animals Co., LTD. [animal license number: SYXK (chuan) 2019-189]. Animal experiments were approved by the Ethics Committee of West China Hospital of Sichuan University. The mice were randomly divided into three groups: control group, NC-mimic group and miR-539-5p-mimic group. Nalm-6 cells in the logarithmic growth phase were selected, and NOD/SCID mice were injected with 5 × 10^6^ Nalm-6 cells, NC-mimic or miR-539-5p-mimic transfected Nalm-6 cells via tail vein, respectively.

### Wright-Giemsa staining

After the bone marrow of mice was taken for smear, 2–3 drops of Wraith-Giemsa dye solution (cat. no. CR2012035; Servicebio, China) was added to dye for 2 min, then the same amount of 0.01 M phosphate buffer (pH6.4–6.8) was stained for 5 min. The morphological changes were observed by a digital slice scanner (Pannoramic 250, 3DHISTECH, Hungary).

### ELISA detection

The expressions of BMP2, IL-10, IL-35, TGF-β1 and TGF-β2 in human/mouse serum and cells were detected by the corresponding ELISA kit (Beyotime, China), each sample was repeated at least three times.

### Real-time quantitative polymerase chain reaction (RT-PCR)

Firstly, total RNA was extracted by Trizol reagent. For miRNA detection, the total RNA was reversed into cDNA by Bulge-LoopTM miRNA qRT-PCR Primer (cat. no. R10031.7; Ribobio, China), and Bulge-Loop miRNA qRT-PCR Starter Kit (cat. no. R11067.2; Ribobio) was used for PCR reaction with 20 µL reaction system. For mRNA detection, the cDNA was obtained by Prime Script RT reagent Kit (cat. no. RR047A; Takara, Japan). Then TB Green Premix Ex Taq Ⅱ (cat. no. RR820A; Takara) was used for PCR reaction. The relative expression levels were calculated using 2^−△△CT^ methods. U6 was used as the internal reference for miRNA expression [20], and β-actin was used for mRNA. The primer sequences were shown in Table 1.

**TABLE 1:** The primer sequences used in the study.

	Gene	Primer
miRNA	hsa-miR-548ba	Bulge-Loop hsa-miR-548ba Primer Set (cat. no. MQPS0001824-1-200; Ribobio, China)
	hsa-miR-1266-5p	Bulge-Loop hsa-miR-1266-5p Primer Set (cat. no. MQPS0000526-1-200; Ribobio, China)
	hsa-miR-466	Bulge-Loop hsa-miR-466 Primer Set (cat. no. MQPS0001494-1-200; Ribobio, China)
	hsa-miR-551a	Bulge-Loop hsa-miR-551a Primer Set (cat. no. MQPS0001855-1-200; Ribobio, China)
	hsa-miR-4484	Bulge-Loop hsa-miR-4484 Primer Set (cat. no. MQPS0001400-1-200; Ribobio, China)
	hsa-miR-1-3p	Bulge-Loop hsa-miR-1-3p Primer Set (cat. no. MQPS0000625-1-200; Ribobio, China)
	hsa-miR-133a-3p	Bulge-Loop hsa-miR-133a-3p Primer Set (cat. no. MQPS0000607-1-200; Ribobio, China)
	hsa-miR-1250-5p	Bulge-Loop hsa-miR-1250-5p Primer Set (cat. no. MQPS0000499-1-200; Ribobio, China)
	hsa-miR-539-5p	Bulge-Loop hsa-miR-539-5p Primer Set (cat. no. MQPS0001793-1-200; Ribobio, China)
	hsa-miR-11401	*Homo sapiens* miR-11401 stem-loop (cat. no. HmiRQP4695; FulenGen, China)
	hsa-miR-6718-5p	*Homo sapiens* miR-6718-5p stem-loop (cat. no. HmiRQP2994; FulenGen, China)
mRNA	TGF-β	F: 5′-GCC​GAC​TAC​TAC​GCC​AAG​GAG​GTC​A-3′
		R: 5′-CGG​TTG​CTG​AGG​TAT​CGC​CAG​GAA​TT-3′
	BMP2	F: 5′-GAC​GTT​GGT​CAA​CTC​TGT​TAA​C-3′
		R:5′-GTCAAGGTACAGCATCGAGATA-3′
	IL-10	F: 5′-GTT​GTT​AAA​GGA​GTC​CTT​GCT​G-3′
		R: 5′-TTC​ACA​GGG​AAG​AAA​TCG​ATG​A-3′
	IL-35	F: 5′-CCT​TGC​ACT​TCT​GAA​GAG​ATT​G-3′
		R: 5′-GGT​CTC​TCT​GGA​ATT​TAG​GCA​A-3′
	Foxp3	F: 5′- AGG​CTC​CAG​AGA​AGC​AGC​GGA​CAC​T-3′
		R: 5′-TCA​GGT​TGT​GGC​GGC​TGG​CGT​TCT​T-3′
	β-actin	F: 5′-GAA​GAT​CAA​GAT​CAT​TGC​TCC-3′
		R: 5′-TAC​TCC​TGC​TTG​CTG​ATC​CA-3′

### Western blot analysis

Total protein was extracted using protein lysate (cat. no. P0013; Beyotime, China), and quantified by BCA protein kit (cat. no. P0009; Beyotime). After denaturation, the total protein was separated by SDS-PAGE and transferred to the PVDF membrane (cat. no. ISEQ00010; Sigma-Aldrich, United States). The PVDF membranes were blocked with 5% skim milk for 2 h, then incubated with the corresponding primary antibody at 4°C overnight, and the secondary antibody at room temperature for 2 h for immune response. The protein was visualized by ECL luminescent solution (cat. no. KF001; Affinity, United States) through the chemiluminescence gel imager (Tanon, China). The primary antibodies BMP2 (cat. no. AF5163), PCAN (cat. no. DF10168), p38 (cat. no. AF6456) and p-p38 (cat. no. AF4001) were purchased from Affinity (United States); Bax (cat. no. ab32503), Bcl-2 (cat. no. ab182858), FOXP3 (cat. no. ab215206), Smad1 (cat. no. ab33902), p-Smad1 (cat. no. ab226821), Smad2 (cat. no. ab33875) and p-Smad2 (cat. no. ab188334) were purchased from Abcam (United Kingdom); ERK1/2 (cat. no. 11257-1-AP), JNK1/2 (cat. no. 66210-1-lg) and p-JNK1/2 (cat. no. 80024-1-RR) were purchased from Proteintech (United States); p-ERK1/2 (cat. no. AP0472) and β-actin (cat. no. AC026); were purchased from abclonal (China). Biotinylated goat anti-rabbit IgG (H+L) (cat. no. ab6721) and goat anti-mouse IgG (H+L) (cat. no. ab6789) were purchased from Abcam (United Kingdom).

### Statistical analysis

All statistical results were analyzed using SPSS version 29.0 software package (IBM Corp., Armonk, NY United States). The Shapiro-Wilk test was used to check whether the data conform to the normal distribution. The data conforming to the normal distribution were expressed as mean ± standard deviation. And the difference between the two groups was analyzed by student's t-test, and the difference among multiple groups was analyzed by one-way analysis of variance. *p* < 0.05 was considered statistically significant.

## Results

### Transcriptome sequencing of PBMCs in B-ALL

**FIGURE 1:**
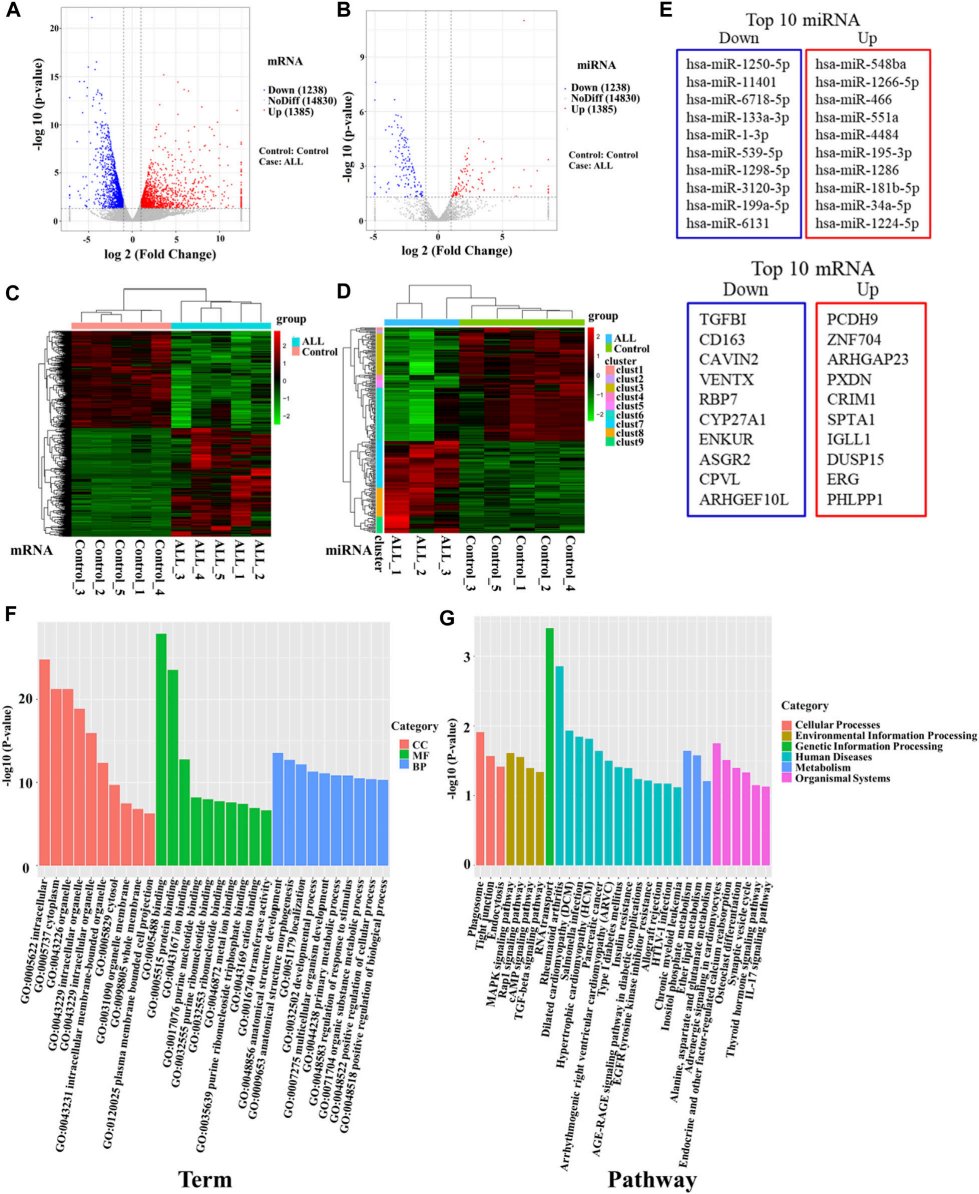
High-throughput sequencing was used to screen differentially expressed genes between B-ALL and controls. **(A)** Volcano map of differentially expressed mRNAs. **(B)** Volcano map of differentially expressed miRNAs. Red dots indicate upregulated genes, blue dots indicate downregulated genes. **(C)** Cluster analysis of differentially expressed mRNAs. **(D)** Cluster analysis of differentially expressed miRNAs. Horizontal represents genes, and each column is a sample. Red indicates high expression and green indicates low expression. **(E)** The top 10 differentially up-regulated and down-regulated miRNAs and mRNAs. **(F)** Bar chart of GO enrichment analysis of differentially expressed miRNAs. **(G)** Bar graph of KEGG pathway enrichment analysis of differentially expressed miRNAs. GO: gene ontology; KEGG: Kyoto Encyclopedia of Genes and Genomes.

The expression characteristics of mRNA and miRNA in PBMCs were analyzed by microarray sequencing. In this study, five samples from the B-ALL and control groups were used for transcriptome mRNA expression analysis. Since only three samples in the B-ALL group were eligible for miRNA analysis, there were three cases in the B-ALL group and five cases in the control group in miRNA analysis. A total of 1,666 miRNA and 17,453 mRNA were obtained in this study. The results of the differential statistics were represented by volcanic maps (Figures 1A, B). Compared with the control group, there were 2,623 differentially expressed mRNAs in the B-ALL group, 1,385 mRNAs were significantly up-regulated and 1,238 were significantly down-regulated (Figure 1A). There were 239 differentially expressed miRNAs, 107 were significantly up-regulated and 132 were significantly down-regulated (Figure 1B). A total of 239 differentially expressed miRNAs were predicted to have a total of 18,319 target genes corresponding to 38,188 target sites. Bidirectional cluster analysis of the differential genes and samples showed that the mRNA and miRNA expression clusters of B-ALL were significantly different from those of the control group (Figures 1C, D). The statistics of the top 10 differentially up-regulated and down-regulated miRNAs and mRNA were shown in Figure 1E.

To further clarify the functions of differentially expressed genes, we conducted an enrichment analysis. The GO enrichment analysis results of differentially expressed miRNA target genes showed that they were mainly enriched in intracellular, cytoplasm, organelle, binding, protein binding, and anatomical structure development, these differentially expressed target genes may play an important role in regulating cell development (Figure 1F). KEGG pathways enrichment were shown in Figure 1G. The results suggest that differentially expressed miRNA target genes had significant differences in regulating cell proliferation, survival and apoptosis, including the TGF-beta signaling pathway, Rap1 signaling pathway, cAMP signaling pathway and MAPK signaling pathway. MiRNAs regulating these pathways may be involved in the abnormal proliferation of B lymphocytes in ALL (Table 2). Among them, the TGF-β signaling pathway has the highest enrichment factor and also has significant differences (*p* < 0.05, Table 2). The involved differentially expressed miRNAs and differentially expressed target genes were analyzed, and miR-539-5p and BMP2 were preliminarily screened (Table 2).

**TABLE 2:** miRNAs and their target genes in KEGG enrichment pathways related to cell proliferation and activation.

KEGG pathway	ID	Rich factor	*p*-value	FDR	miRNA		Target EDGs	
TGF-beta signaling pathway	hsa04350	0.976	0.046	0.68	hsa-miR-3176	up	TGF-β1	down
					hsa-miR-3120-3p	down	TGF-β2	up
					hsa-miR-6852-3p	down	SMAD1	up
					**hsa-miR-539-5p**	**down**	**BMP2**	**up**
Rap1 signaling pathway	hsa04015	0.961	0.028	0.65	hsa-miR-548ba	up	RAP1A	down
					hsa-miR-654-5p	down	RAP1GAP	up
MAPK signaling pathway	hsa04010	0.956	0.025	0.65	hsa-miR-1286	up	RASGRP4	down
					hsa-miR-96-5p	up	RASGRP3	down
cAMP signaling pathway	hsa04024	0.959	0.040	0.65	hsa-miR-1-3p	down	RAPGEF3	up
					hsa-miR-6718-5p	down	ATP2B4	up

The bold values are the miRNA and its target genes screened in this study.

### miR-539-5p/BMP2 was associated with Treg activation in B-ALL

**FIGURE 2:**
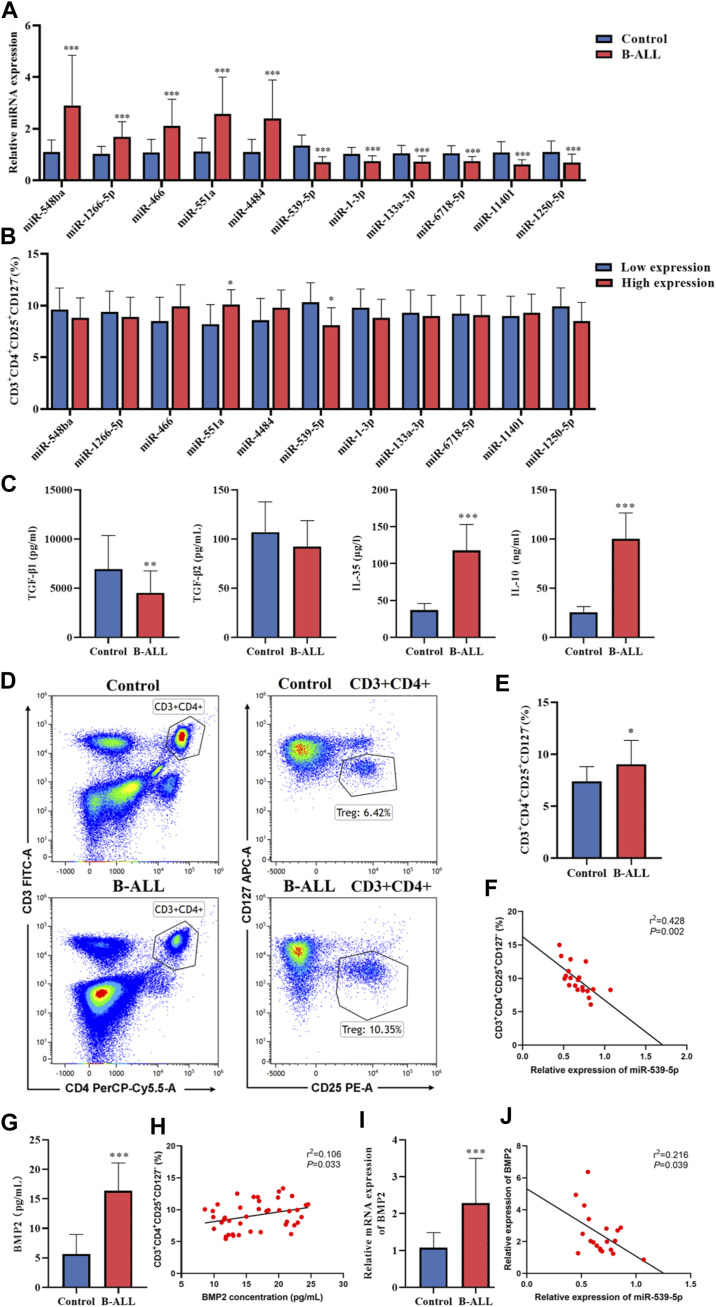
Expression of miR-539-5p/BMP2 in B-ALL and its relationship with Treg activation. **(A)** RT-PCR was used to verify the differential expressed miRNAs in more clinical samples. **(B)** With the median of differentially expressed miRNA expression as the critical value, B-ALL children were divided into low expression group and high expression group, and the proportion of Treg was analyzed. **(C)** The expressions of TGF-β1, TGF-β2, IL-35 and IL-10 in serum were detected by ELISA. **(D)** The proportion of Treg in peripheral blood was detected by flow cytometry. **(E)** Statistical bar graph of flow cytometry. **(F)** Correlation between miR-539-5p expression level and Treg ratio in B-ALL. **(G)** Serum BMP2 content was detected by ELISA. **(H)** Correlation between BMP2 concentration and Treg ratio in B-ALL. **(I)** The gene expression of BMP2 was detected by RT-PCR. **(J)** Correlation between expression levels of miR-539-5p and BMP2 in B-ALL. *p < 0.05, **p < 0.01, ***p < 0.001.

To verify the accuracy of the sequencing data, blood samples were collected from 20 B-ALL patients and 15 controls. The miRNAs with significant expression differences were selected from the sequencing data, among which miR-548ba, miR-1266-5p, miR-466, miR-551a and miR-4484 were the top five miRNAs with up-regulated multiples, miR-539-5p, miR-1-3p, miR-133a-3p, miR-6718-5p, miR-11401 and miR-1250-5p were the top 6 miRNAs that found to be down-regulated, and the results of sequencing data were verified by RT-PCR. As shown in Figure 2A, the RT-PCR results were consistent with the sequencing results (*p* < 0.001). In addition, in order to study the relationship between the differential expression of miRNA and the proportion of Treg in peripheral blood of children with B-ALL, we divided B-ALL children into low expression group and high expression group with the median expression of each differentially expressed miRNA as the critical value, and analyzed whether there was a difference in the proportion of Treg between the low expression group and the high expression group. The results showed that there was no statistical significance in the proportion of Treg between miR-548ba (median: 3.03), miR-1266-5p (median: 1.53), miR-466 (median: 1.81), miR-4484 (median: 2.10), miR-1-3p (median: 0.73), miR-133a-3p (median: 0.68), miR-6718-5p (median: 0.71), miR-11401 (median: 0.59), and miR-1250-5p (median: 0.46) low expression group and high expression group (*p* > 0.05, Figure 2B). Only miR-551a (median: 2.07) and miR-539-5p (median: 0.67) had significant differences in the proportion of Treg cells between the low and high expression groups (*p* < 0.01, Figure 2B). Therefore, we further identified miR-539-5p as the object of follow-up research. Then, the expressions of TGF-β1, TGF-β2, IL-35 and IL-10 in the serum of B-ALL were analyzed by ELISA, which showed that TGF-β1, IL-35 and IL-10 were significantly increased in the B-ALL group (*p* < 0.01, Figure 2C). The proportion of Treg was significantly higher in the B-ALL group than that in the control group (*p* < 0.05, Figures 2D, E). Correlation analysis showed that the proportion of Treg was significantly negatively correlated with the expression of miR-539-5p (Figure 2F). In addition, the expression of BMP2 in serum was analyzed by ELISA, which showed that the level of BMP2 in the B-ALL group was significantly higher than that in the control group (*p* < 0.001, Figure 2G), and the proportion of Treg was significantly positively correlated with the expression of BMP2 (*p* < 0.05, Figure 2H). RT-PCR results also showed that the gene expression of BMP2 in the B-ALL group was significantly higher than that in the control group (*p* < 0.001, Figure 2I), and it was significantly negatively correlated with the gene expression of miR-539-5p (*p* < 0.05, Figure 2J). These results indicated that miR-539-5p could significantly regulate the gene expression and secretion of BMP2 to participate in Treg activation in B-ALL.

### miR-539-5p directly targets BMP2

**FIGURE 3:**
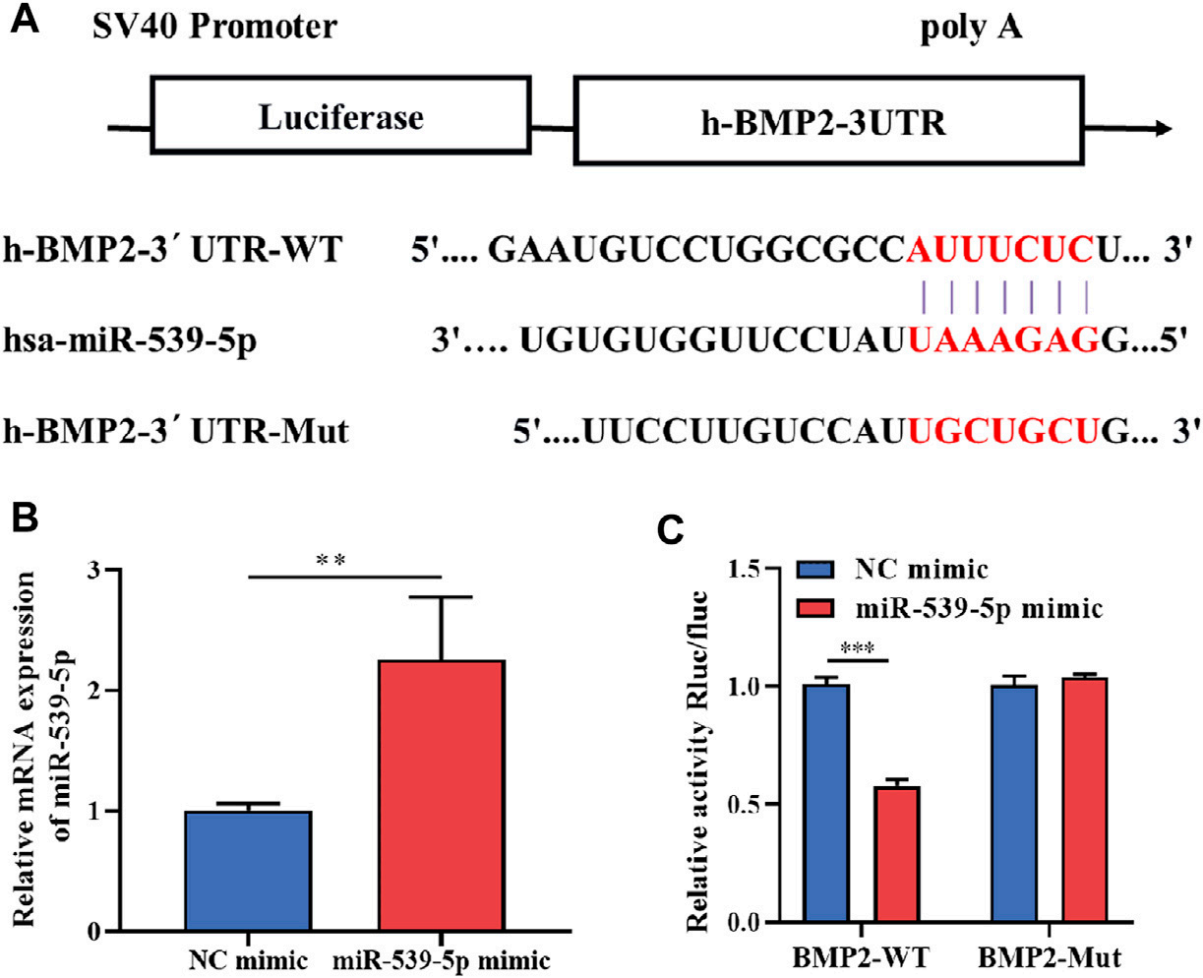
Binding relationship between miR-539-5p and BMP2. **(A)** The predicted binding site of miR-539-5p and BMP2 3′UTR. **(B)** RT-PCR was used to detect the gene expression of miR-539-5p after transfecting NC mimic or mir-539-5p mimic. **(C)** Double luciferase reporter assay to verify the binding relationship between miR-539-5p and BMP2. *p < 0.05, **p < 0.01, ***p < 0.001.

To further clarify whether there is a clear targeting relationship between miR-539-5p and BMP2, TargetScan was used to analyze the possible binding sites between miR-539-5p and BMP2 3′UTR. The result was shown in Figure 3A. After transfecting H293T cells with a 5 pmol NC mimic or miR-539-5p mimic, the gene expression of miR-539-5p was detected by RT-PCR, and results showed that miR-539-5p expression in the miR-539-5p mimic group was significantly increased compared with that of the NC mimic group (*p* < 0.01, Figure 3B). Then, the relationship between miR-539-5p and BMP2 was verified by double luciferase reporter assay (Figure 3C). The results showed that there was no significant difference in luciferase activity between NC-mimic+BMP2-Mut and miR-539-5p-mimic+BMP2-Mut groups (*p* > 0.05), while the luciferase activity was significantly decreased in the miR-539-5p-mimic+BMP2-WT group compared with NC-mimic+BMP2-WT group (*p* < 0.001), indicating that miR-539-5p directly targeted the 3′UTR region of BMP2, thereby affecting BMP2 gene expression.

### Effects of miR-539-5p/BMP2 on proliferation and apoptosis of Nalm-6 cells

**FIGURE 4:**
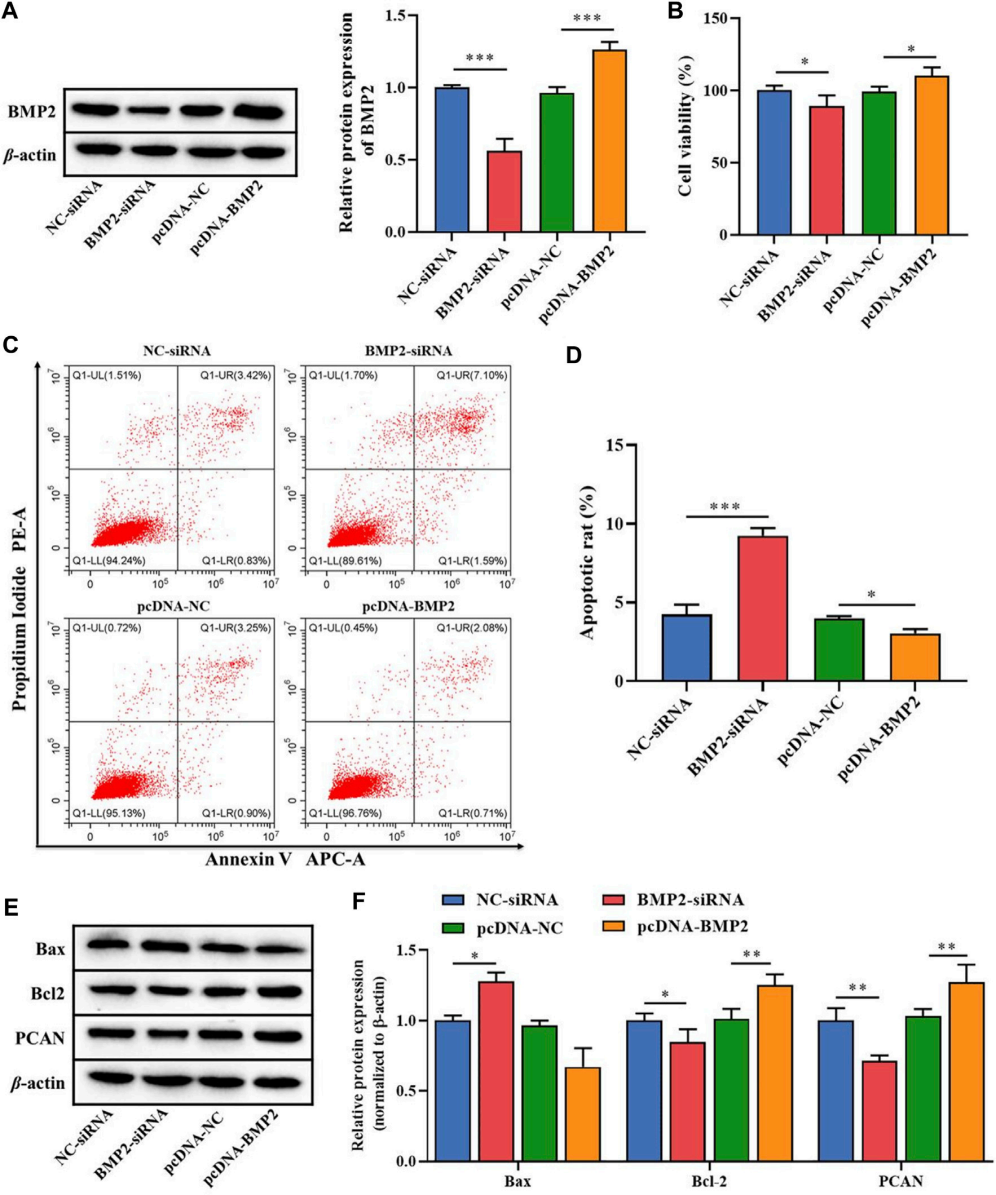
Effects of miR-539-5p downstream gene BMP2 on proliferation and apoptosis of Nalm-6 cells. **(A)** After transfection with BMP2-siRNA or pcDNA-BMP2, the protein expression of BMP2 was detected by WB to verify the transfection efficiency. **(B)** CCK8 was used to detect the viability of Nalm-6 cells after intervention or overexpression of BMP2. **(C)** The apoptosis of Nalm-6 cells was detected by flow cytometry. **(D)** Histogram of percentage of apoptotic cells. **(E)** The protein expressions of Bax, Bcl-2 and PCAN were detected by WB. **(F)** Histogram of gray level analysis of protein bands. *p < 0.05, **p < 0.01, ***p < 0.001.

To clarify the role of the downstream target gene BMP2 in B-ALL cells, BMP2-siRNA and pcDNA-BMP2 were constructed to transfect Nalm-6 cells. The transfection efficiency analysis by WB showed that the protein expression of BMP2 was significantly reduced in the BMP2-siRNA group compared with the NC-siRNA, and was significantly increased in the pcDNA-BMP2 group compared with the pcDNA-NC group (*p* < 0.001, Figure 4A). In addition, compared with NC-siRNA or pcDNA-NC group, the BMP2-siRNA group significantly inhibited cell proliferation activity (*p* < 0.05, Figure 4B), promoted cell apoptosis (*p* < 0.001, Figures 4C, D), increased the protein expression of Bax, and decreased the protein expression of Bcl-2 and PCN2 (*p* < 0.05, Figures 4E, F), while pcDNA-BMP2 group showed the opposite effect (Figures 4B–F). These results suggested that BMP2, as the downstream target gene of miR-539-5p, could significantly promote cell viability and inhibit apoptosis in Nalm-6 cells.

**FIGURE 5:**
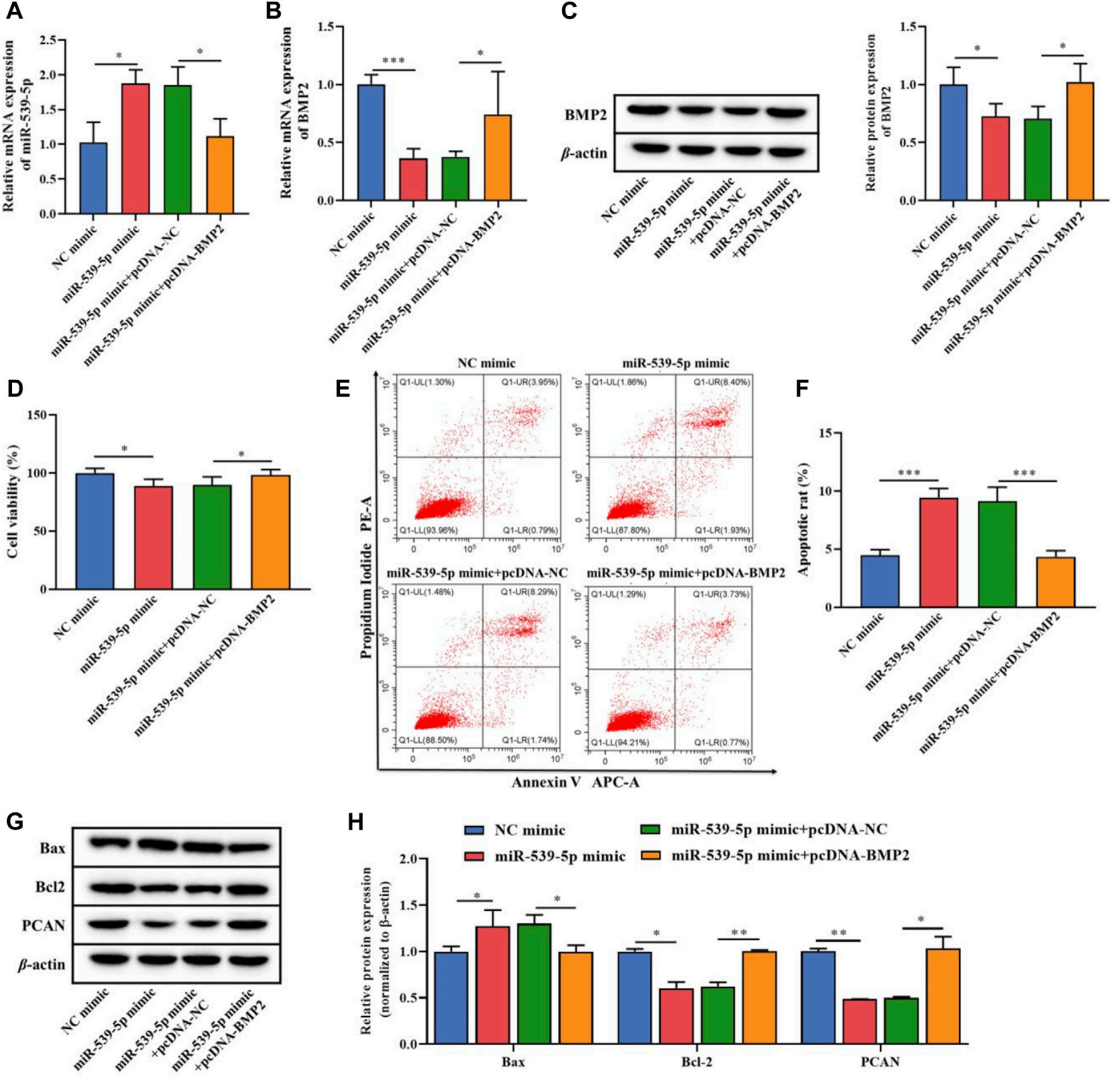
Effects of miR-539-5p/BMP2 regulatory axis on proliferation and apoptosis of Nalm-6 cells. After transfection of Nalm-6 cells with miR-539-5p-mimic and/or pcDNA-BMP2, the gene expression of miR-539-5p **(A)** and BMP2 **(B)** was detected by RT-PCR. **(C)** The protein expression of BMP2 was detected by WB. **(D)** CCK8 was used to detect the viability of Nalm-6 cells. **(E)** The apoptosis of Nalm-6 cells was detected by flow cytometry. **(F)** Histogram of percentage of apoptotic cells. **(G)** The protein expressions of Bax, Bcl-2 and PCAN were detected by WB. **(H)** Histogram of gray level analysis of protein bands. *p < 0.05, **p < 0.01, ***p < 0.001.

On this basis, we further analyzed the effects of miR-539-5p regulation of BMP2 on the proliferation and apoptosis of Nalm-6 cells. MiR-539-5p mimic and/or pcDNA-BMP2 were used to co-transfect Nalm-6 cells, and RT-PCR analysis showed that compared with the NC-mimic group, miR-539-5p-mimic significantly increased the gene expression of miR-539-5p (*p* < 0.05, Figure 5A). In addition, the expression analysis of BMP2 by RT-PCR and WB showed that miR-539-5p-mimic significantly decreased the gene and protein expressions of BMP2 compared with the NC-mimic group (*p* < 0.05, Figures 5B, C). And compared with the miR-539-5p-mimic+pcDNA-NC group, miR-539-5p-mimic+pcDNA-BMP2 significantly decreased the gene expression of miR-539-5p (*p* < 0.05, Figure 5A), and promoted the gene and protein expression of BMP2 (*p* < 0.05, Figures 5B, C). Analysis of the proliferation and apoptosis activity of Nalm-6 cells showed that miR-539-5p-mimic significantly decreased the cell viability (*p* < 0.05, Figure 5D), promoted apoptosis (*p* < 0.001, Figures 5E, F), increased the protein expression of Bax, and decreased the protein expression of Bcl-2 and PCN2 compared with NC mimic group (*p* < 0.05, Figures 5G, H). Compared with the miR-539-5p-mimic+pcDNA-NC group, miR-539-5p-mimic+pcDNA-BMP2 significantly reversed the effects of miR-539-5p-mimic on Nalm-6 cells. These results suggested that miR-539-5p might inhibit Nalm-6 cell proliferation and promote apoptosis by targeting BMP2.

### Nalm-6 cell synergies with TGF-β1 to promote Treg activation through TGF-β1/Smads/MAPK pathways

**FIGURE 6:**
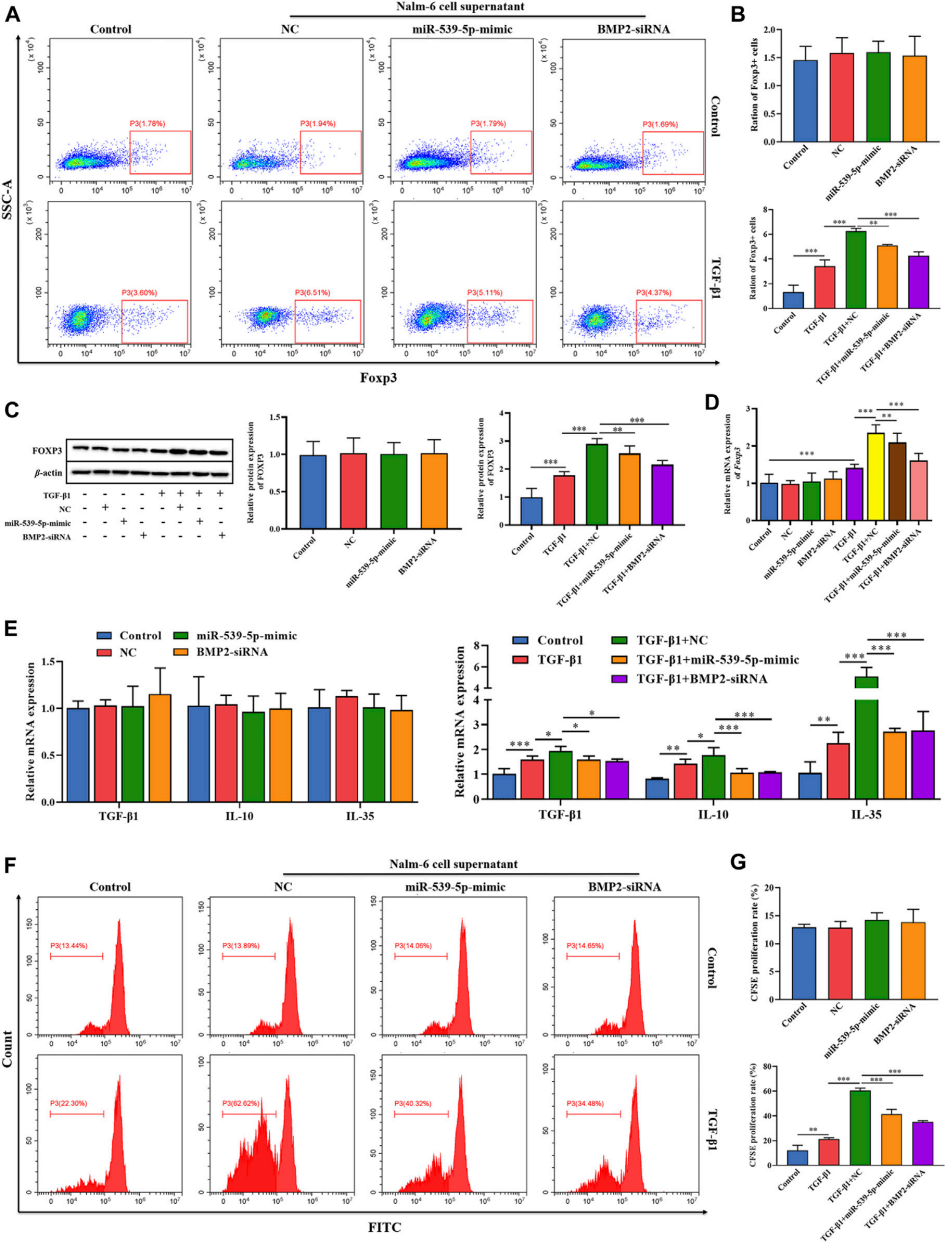
Effects of miR-539-5p/BMP2 in Treg activation induced by Nalm-6 cells. **(A)** The effect of different induction methods on the proportion of Foxp3^+^ was detected by flow cytometry. **(B)** Statistical results of Foxp3^+^ cell percentage. **(C)** The effect of different induction methods on Foxp3 protein expression was detected by WB. **(D)** Foxp3 gene expression was detected by RT-PCR. **(E)** The effects of different induction methods on the gene expressions of TGF-β1, IL-10 and IL-35 were detected by RT-PCR. **(F)** The proliferation activity was analyzed by CFSE. **(G)** Statistical results of CFSE proliferation rate. *p < 0.05, **p < 0.01, ***p < 0.001.

To explore the effects of Nalm-6 cells on Treg activation, the culture supernatant of Nalm-6 cells was collected and incubated with human peripheral blood CD4^+^CD25^-^T cell induction medium at a ratio of 1:1 to stimulate Treg differentiation. The results showed that in the absence of TGF-β1 stimulation, the culture supernatant of Nalm-6 cells treated with different treatments could not directly induce the expression of Foxp3 in CD4^+^CD25^-^T cells (*p* > 0.05, Figures 6A–D). Compared with the control group, TGF-β1 significantly induced the Foxp3^+^ proportion (Figures 6A, B) and Foxp3 expression (Figures 6C, D) in CD4^+^CD25^-^T cells (*p* < 0.001), and also increased the mRNA expression levels of cytokines TGF-β1, IL-10 and IL-35 (*p* < 0.01, Figure 6E). When simultaneously stimulated with TGF-β1, the culture supernatant of Nalm-6 cells could further enhance the promoting effects of TGF-β1, showing a synergistic effect with TGF-β1 (*p* < 0.001, Figures 6A–D). In addition, the supernatant of miR-539-5p-mimic and BMP2-siRNA transfected cells attenuated the synergistic effect between the Nalm-6 cell supernatant and TGF-β1 (*p* < 0.001, Figures 6A–D). Similarly, in cell proliferation experiments, we also found that the culture supernatant of Nalm-6 cells in each group could promote the proliferation of CD4^+^CD25^-^T cells induced by TGF-β1, showing a synergistic effect with TGF-β1 (*p* < 0.001, Figures 6F, G). However, after transfected with miR-539-5p-mimic or BMP2-siRNA, the synergistic effect between the Nalm-6 cells and TGF-β1 decreased (*p* < 0.001, Figures 6F, G). These experiments indicated that Nalm-6 cells could cooperate with TGF-β1 to stimulate CD4^+^CD25^-^T cells to differentiate into Foxp3^+^Treg cells, which might be achieved by inhibiting miR-539-5p to promote BMP2 secretion.

**FIGURE 7:**
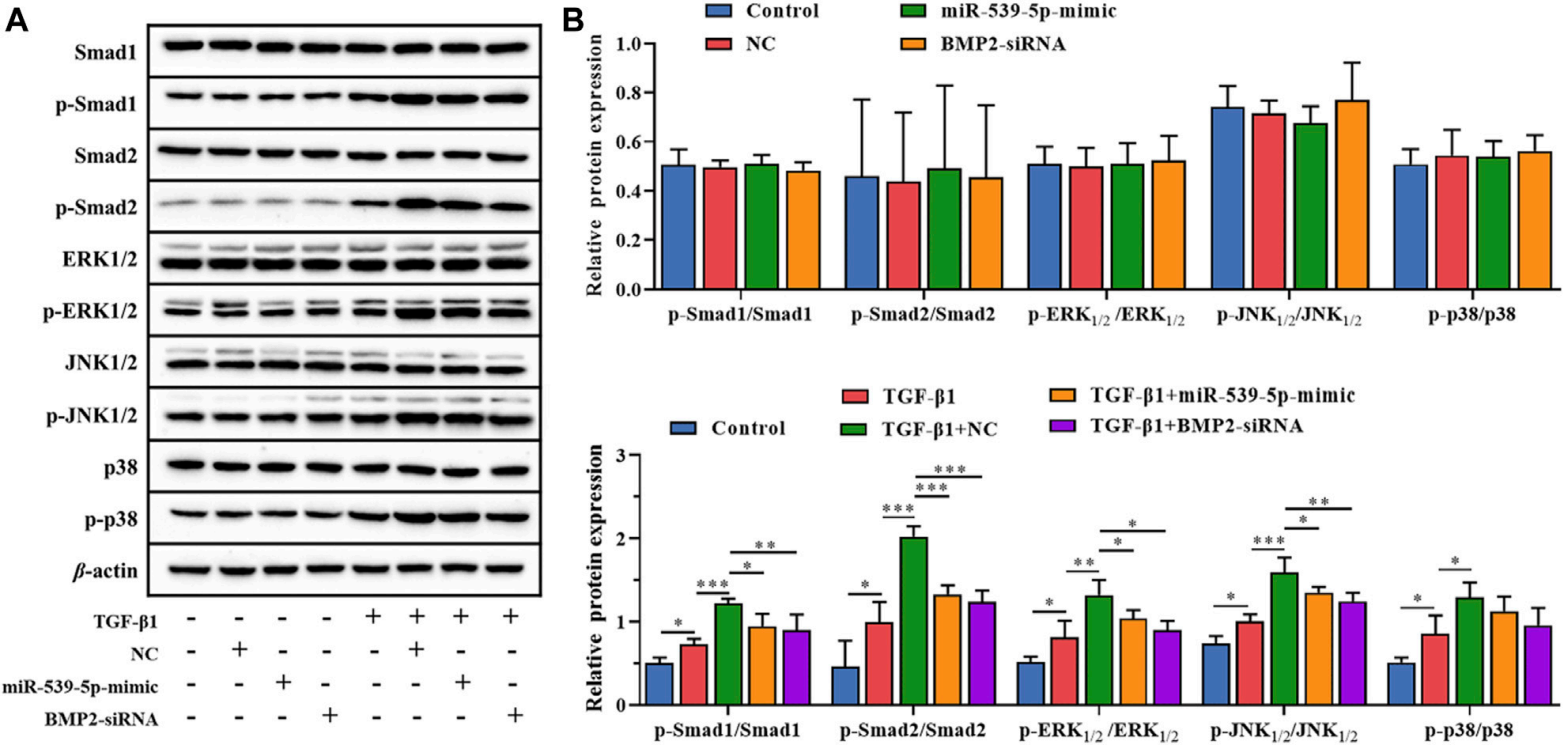
Effects of miR-539-5p/BMP2 on TGF-β/Smads/MAPK pathway in Treg induced by Nalm-6 cells. **(A)** The protein expressions of Smad1, p-Smad1, Smad2, p-Smad2, ERK1/2, p-ERK1/2, JNK1/2, p-JNK1/2, p38 and p-p38 were detected by WB. **(B)** Histogram of gray level analysis of protein bands. *p < 0.05, **p < 0.01, ***p < 0.001.

In order to further clarify the downstream signaling pathway involved in Nalm-6 cells synergism with TGF-β1, the protein expressions of TGF-β/Smads/MAPK pathway in different treatment groups were detected (Figure 7). The results showed that compared with the control group, the supernatant of Nalm-6 cells alone could not promote the phosphorylation of Smad1, Smad2, ERK1/2, JNK and p38 in CD4^+^CD25^-^T cells (*p* > 0.05). TGF-β1 could significantly promote the phosphorylation of Smad1, Smad2, ERK1/2, JNK and p38 (*p* < 0.05). The induction and pro-phosphorylation effects were strongest when the supernatant of Nalm-6 cells was co-treated with TGF-β1 (*p* < 0.05). However, when TGF-β1 was treated with miR-539-5p-mimic and BMP2-siRNA supernatant, the induction and pro-phosphorylation effects were decreased, but still higher than those of the control group (*p* < 0.001). In conclusion, Nalm-6 cells and TGF-β1 cooperatively regulate TGF-β/Smads/MAPK pathways to promote Treg activation.

### Effects of miR-539-5p/BMP2 on Treg induction in B-ALL mice

**FIGURE 8:**
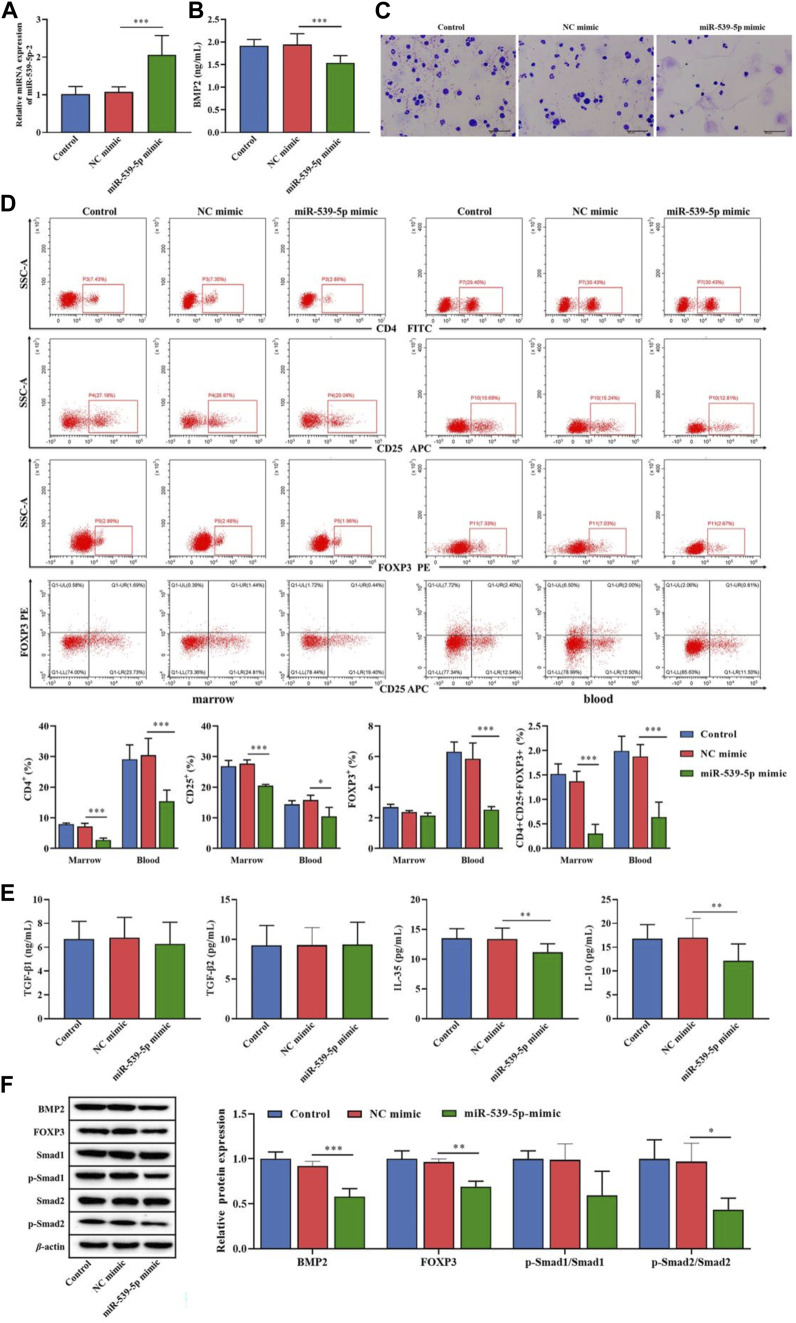
Effects of miR-539-5p/BMP2 on B-ALL mice *in vivo*. **(A)** RT-PCR was used to detect the expression of miR-539-5p in mouse PBMCs. **(B)** The content of BMP2 in plasma was detected by ELISA. **(C)** The cytopathic changes in bone marrow smears were observed by Wright-Giemsa staining. **(D)** The proportion of CD4^+^CD25^+^Foxp3^+^Treg cells in the bone marrow and peripheral blood was detected by flow cytometry. **(E)** The expression of TGF-β1, TGF-β2, IL-35 and IL-10 in plasma was detected by ELISA. **(F)** The protein expressions of BMP2, FOXP3, Smad1, p-Smad1, Smad2 and p-Smad2 in bone marrow were detected by WB. *p < 0.05, **p < 0.01, ***p < 0.001.

We further used Nalm-6 cells to construct the B-ALL model in NOD/SCID mice, and explored the effect of miR-539-5p/BMP2 on Treg activation *in vivo*. Firstly, analysis of the expression levels of miR-539-5p and BMP2 in PBMCs showed that in the miR-539-5p-mimic group, the mRNA expression level of miR-539-5p was significantly increased (*p* < 0.001, Figure 8A), and the level of BMP2 was significantly decreased (*p* < 0.001, Figure 8B). Overexpression of miR-539-5p also inhibited BMP2 levels in mice. Then, the Wraith-Giemsa staining results of mouse bone marrow tissues were shown in Figures 8A, C large number of abnormal lymphocytes were observed in the control and NC-mimic groups, with large volume, dysregulated nucleo-plasmic ratio, basophilic cytoplasm, and large and hyperchromatic nuclei. In the miR-539-5p-mimic group, no obvious abnormalities were observed in all types of cells under the microscope. Furthermore, analysis of the proportions of CD4^+^CD25^+^Foxp3^+^Treg showed that the proportions of CD4^+^, CD25^+^ and CD4^+^CD25^+^Foxp3^+^ in the bone marrow and peripheral blood of the miR-539-5p-mimic group were significantly lower than those of the NC-mimic group (*p* < 0.001, Figure 8D). ELISA assay of serum cytokine levels showed that the levels of IL-35 and IL-10 in miR-539-5p-mimic were significantly lower than those in the NC-mimic group (*p* < 0.001), but there was no significant difference in TGF-β1 and TGF-β2 among all groups (*p* > 0.05, Figure 8E). WB assay showed that miR-539-5p-mimic reduced the levels of BMP2, Foxp3 and p-Smad2/Smad2 (*p* < 0.05, Figure 8F). *In vivo* experiments confirmed that miR-539-5p-mimic could inhibit BMP2 secretion and Treg differentiation in B-ALL model mice.

## Discussion

As an important regulatory factor of genes, miRNAs participate in physiological and pathological processes *in vivo*, and their abnormal expression is closely related to cancers [21]. In recent years, a large number of studies have confirmed that miRNA can directly participate in the cancer process [22], and can be used for the early clinical diagnosis, prognosis evaluation, chemotherapy drug resistance evaluation and toxicity prediction of cancers [23, 24]. Similarly, miRNAs can directly participate in ALL as proto-oncogenes or tumor suppressor genes [25], and may become an effective indicator related to B-ALL. In this study, the significantly low expression of miR-539-5p and its target gene BMP2 in B-ALL were screened out by high-throughput sequencing. Functional enrichment analysis showed that the main enrichment pathway involved the TGF-β pathway. Further analysis showed that miR-539-5p and BMP2 were significantly correlated with the proportion of Treg in B-ALL. *In vitro* and *in vivo* experiments confirmed that the low expression of miR-539-5p in B-ALL could directly target and promote BMP2 expression, and the increased BMP2 could further promote the proliferation and inhibit apoptosis of Nalm-6 cells, and cooperate with TGF-β1 to stimulate the differentiation of human CD4^+^CD25^-^T cells into Treg. TGF-β1/Smads/MAPK pathway played an important role in the regulation of Treg activation by miR-539-5p/BMP2.

MiRNA is a class of endogenous non-coding single-stranded small RNA, which is involved in the regulation of post-transcriptional gene expression by specifically binding to the target genes [26, 27]. MiR-539 has been confirmed as a suppressor factor in cancers, and plays an inhibitory role in a variety of cancers, including breast cancer [28], osteosarcoma [29], and pancreatic cancer [30]. However, there are no relevant research reports on miRNA-539-5p in B-ALL at present. Our study confirmed that the expression of miR-539-5p was significantly decreased in B-ALL, which may play a role in cancer suppression. There was a targeting site between miR-539-5p and BMP2, and miR-539-5p could negatively target the expression of BMP2 in B-ALL. BMP is a key regulator of various developmental stages and plays an important role in biological processes [31]. Studies have shown that BMP2 has a dual effect on tumorigenesis, which may promote [32–34] or inhibit [35] cancer progression. This study confirmed that BMP2 was significantly increased in B-ALL, and the high expression of BMP2 promoted the proliferation and inhibited the apoptosis of Nalm-6 cells, significantly decreased the expression of Bax, and increased the expression of Bcl-2 and PCNA. Bax is a regulator of Bcl-2 activity, both Bcl-2 and Bax play an important role in cell apoptosis [36, 37], and Bcl-2 is increased in most cancers, while Bax is decreased [38]. PCNA is a protein expressed in a specific phase of the cell cycle and is generally considered to be positively correlated with cancer cell proliferation [39]. In this study, further experiments suggested that miR-539-5p could regulate the proliferation and apoptosis of B-ALL cells by targeting BMP2.

BMP has been shown to promote T cell activation, and regulate the proliferative response of naive CD4+T cells [40]. When BMP signaling is inhibited, it leads to impaired maturation, differentiation and function of Foxp3^+^ Treg [41]. Similarly, miRNA has also been shown to inhibit the function of immune cells and promote immune escape by regulating the proliferation and differentiation of immune cells, and the secretion of effectors in the tumor microenvironment [42]. The analysis of this study showed that the significantly low expression of miR-539-5p and its target gene BMP2 in B-ALL were both significantly correlated with the percentage of Treg in B-ALL. Treg cells, a subset of T cells, play a critical role in maintaining the homeostasis of the immune system [43]. In the tumor microenvironment, Treg can inhibit anti-tumor immune response and promote tumor immune escape, thus affecting the development of tumors [44]. The inhibitory adaptive immune response induced by Treg is mediated through the expression of TGF-β1 [45]. The inhibition ability of Treg induced by BMP2 and TGF-β1 is stronger than that induced by TGF-β1 alone [46]. In this study, we found that the culture supernatant of Nalm-6 cells could cooperate with TGF-β1 to promote the proportion of Foxp3+Treg and increase the expression of Foxp3 in CD4^+^CD25^-^T cells. Transfection with miR-539-5p-mimic and BMP2-siRNA attenuated the synergistic effect of Nalm-6 cells and TGF-β1 on Foxp3 promotion. Studies have confirmed that Foxp3+Treg could be used as a therapeutic target to promote anti-tumor immunity [47]. Once Foxp3+Treg loses its transcription factor Foxp3, it will lose immunomodulatory function [48]. A recent relevant study recorded a slight insignificant increase in Treg cells in children with ALL compared to controls, and the increase in Treg appeared to occur due to a decrease in miR-155 [49]. A large number of studies have confirmed that miRNAs play an important role in the alteration of Treg in B-ALL [50]. Our study confirmed that miR-539-5p can regulate BMP2 to participate in Treg activation in B-ALL.

BMP belongs to the TGF-β superfamily, and a large number of studies have confirmed that TGF-β plays an important role in the inhibitory activity of CD4^+^CD25^+^T cells [51]. This study showed that the target genes of differentially expressed miR-539-5p in B-ALL were mainly enriched in the TGF-β pathway. TGF-β pathway is involved in cancer cell proliferation, differentiation, apoptosis and other functions [52]. When TGF-β binds specifically to the receptor, it initiates both classical Smad and non-Smad pathways [53]. MAPK pathway includes ERK, JNK and p38, which are the main signaling pathway for TGF-β to initiate non-Smad in cells [54]. Both Smad and non-Smad pathways are involved in the evolution of Treg differentiation induced by TGF-β [55]. This study found that the culture supernatant of Nalm-6 cells in collaboration with TGF-β1 could activate the phosphorylation of Smad1, Smad2, ERK, JNK and p38. In addition, the transfection of miR-539-5p-mimic and BMP2-siRNA inhibited the activation of the TGF-β1/Smads/MAPK pathway induced by Nalm-6 cells. These results suggested that the regulatory effect of Nalm-6 cells on Treg is achieved through the negative regulation of BMP2 by miR-539-5p to regulate the TGF-β1/Smads/MAPK pathway.

## Conclusion

In conclusion, this study showed that miR-539-5p was one of the significantly down-regulated miRNAs in B-ALL and was significantly correlated with Treg expression, and BMP2 was the most differential expressed gene among miR-539-5p target genes, which was mainly enriched in the TGF-β pathway. The expression levels of miR-539-5p and BMP2 were both significantly correlated with the proportion of Tregs. *In vitro* and *in vivo* experiments confirmed that miR-539-5p could directly target BMP2, and the low expression of miR-539-5p in B-ALL resulted in significantly increased levels of BMP2. High BMP2 not only promoted Nalm-6 cell proliferation and inhibited apoptosis, but also cooperated with TGF-β1 to promote the expression of transcription factor Foxp3 to promote the differentiation of CD4^+^CD25^-^T cells into Foxp3^+^Treg through the TGF-β1/Smads/MAPK pathways. This study demonstrated that miR-539-5p targeting BMP2 was involved in the Treg activation through the TGF-β1/Smads/MAPK pathway and played an important role in B-ALL.

## Data Availability

The original contributions presented in the study are included in the article/Supplementary Material, further inquiries can be directed to the corresponding author.

## Footnotes

1
www.targetscan.org
