# Supplementary material for: miR-539-5p targets BMP2 to regulate Treg activation in B-cell acute lymphoblastic leukemia through TGF-β/Smads/MAPK
Source: Exp Biol Med (Maywood). 2024 Feb 13;249:10111. doi: 10.3389/ebm.2024.10111 (PMC10954254; doi:10.3389/ebm.2024.10111)
Supplement: Supplementary file 1 [file Table1.pdf]

Supplement Table 1. Basic information of 3 B-ALL samples in miRNA sequencing

Sample	Treg ($CD4^{+}CD25^{+}CD127^{-}$)	FAB typing	Immune typing	Fusion gene
B-ALL 1	9.62%	L2	Pre-B	E2A-PBX1
B-ALL 2	10.79%	L2	early Pre-B	negative
B-ALL 3	10.87%	L2	early Pre-B	TEL-AML1
